# Harnessing mass spectrometry-based proteomics for continuous directed evolution

**DOI:** 10.1093/synbio/ysaf017

**Published:** 2025-12-04

**Authors:** Katharina Belt, David Obe, Mark A Wilson, A Harvey Millar, Ulschan Bathe

**Affiliations:** ARC Centre of Excellence in Plant Energy Biology, School of Molecular Sciences, University of Western Australia, Crawley WA 6009, Australia; Department of Biochemistry and Redox Biology Center, University of Nebraska, Lincoln, NE 68588, USA; Department of Biochemistry and Redox Biology Center, University of Nebraska, Lincoln, NE 68588, USA; ARC Centre of Excellence in Plant Energy Biology, School of Molecular Sciences, University of Western Australia, Crawley WA 6009, Australia; Biotechnology of Horticultural Crops, TUM School of Life Sciences, Technical University of Munich, Liesel-Beckmann-Str. 1, Freising 85354, Bavaria, Germany

**Keywords:** spectrometry-based proteomics, continuous directed evolution, OrthoRep, Arabidopsis methionine synthase, enzyme longevity

## Abstract

Continuous directed evolution is a powerful Synthetic Biology tool to engineer proteins with desired functions *in vivo*. Mimicking natural evolution, it involves repeated cycles of high-frequency mutagenesis, selection, and replication within platform cells, where the function of the target gene is tightly linked to the host cell’s fitness. However, cells might escape the selection pressure due to the inherent flexibility of their metabolism, which allows for adaptation. Whole-proteome analysis as well as targeted proteomics offer valuable insights into global and specific cellular changes. They can identify modifications in the target protein and its interactors to help understand its evolution and network integration. Using the continuous evolution of the *Arabidopsis thaliana* methionine synthases AtMS1 and AtMS2 as an example, we show how mass spectrometry-based proteomics was able to assess the abundance of target enzymes, identify flaws in population construction, measure methionine metabolic adaptation, and allow informed decision-making in the evolution campaign.

## Graphical Abstract

**Graphical Abstract:**
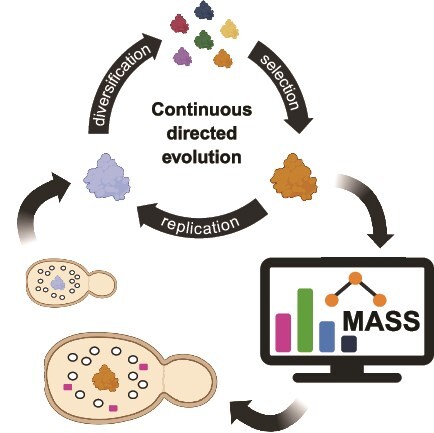

## Introduction

Directed evolution is one of Synthetic Biology’s most outstanding achievements. It aims at engineering biomolecules, e.g. enzymes, with required phenotypes or functions [1]. Classical directed evolution was awarded the 2018 Nobel Prize [2], but continuous directed evolution (CDE) is considered even more powerful overcoming limitations in scale of experimentation and depth of evolutionary search. Thus, it holds the potential to shape future medicine, agriculture and industry.

The principle of CDE is *in vivo* sequence diversification, selection and replication, and couples the function of the target gene to the host cell’s fitness [3]. Diverse CDE tools allow directed evolution in different platform cell types, e.g. phage-based, bacterial, yeast or mammalian systems [4–10] to engineer antibodies, substrate specificity, improved catalytic efficiency, and novel enzyme activities [11–14].

**Figure 1:**
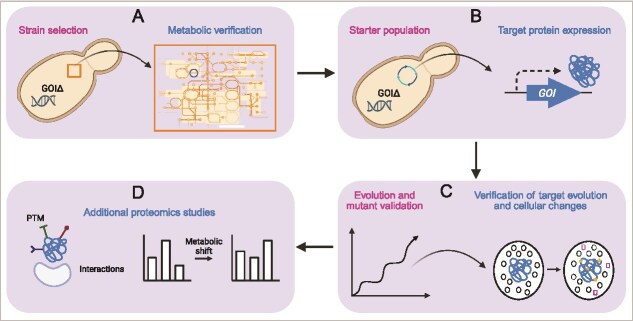
Proteomics in continuous directed evolution. Proteomic can be incorporated at critical stages of CDE to evaluate protein-level changes (A-D). A. The CDE workflow begins with baseline proteomic analysis of the initial strain to assess global protein expression. B. Targeted proteomics can validate expression and abundance of the protein of interest. C. Untargeted proteomic analysis can identify early protein shifts in CDE, e.g. protein-level adaptations. The best-performing strains are finally selected, and proteomic validation confirms the protein function associated with the observed traits (D). The figure was created in Biorender, Bathe, U. (2025) https://BioRender.com/rlfhguw.

CDE is considered powerful because repeated cycles of mutagenesis and screening inside the platform organism allow efficient and scalable evolution. However, the host cells’ metabolism is a complex and interconnected protein machinery, and CDE may cause trade-off effects in the proteome because the selection pressure is not selective enough or potentially affects other pathways. In the worst case, the platform cells escape the selection pressure entirely. In such cases, post-CDE evaluation would reveal that evolved phenotypes are not only or not exclusively connected to the target gene’s function. To reduce the risk of an unsuccessful campaign and to make CDE even more powerful, whole-system analysis might be used to look beyond single target genes, transcripts, and metabolites. While whole genome and transcriptome sequencing would give some upstream insights into a cell’s real condition, metabolomics and proteomics deliver more information about the platform’s cellular changes because they are closer to the phenotype. Proteomics is not utilized as a standard tool in SynBio experiments thus far [15]; for example, we are aware of only one case of a CDE study utilizing whole-cell proteomics [16]. Yet, mass spectrometry-based proteomics can provide qualitative and quantitative insights into an organism’s entire proteome or of specific protein targets and their relative or absolute abundance [17–19] (Fig. 1). For example, proteomics data can help to validate the platform strain (Fig. 1A) and the target protein expression (Fig. 1B and C), directly show interaction of the engineered target with other biomolecules and pathways, and potential trade-offs (Fig. 1C and D). These attributes make proteomics an invaluable tool for CDE, enabling the verification of intermediate steps, validation of final functions, and troubleshooting of challenges during the evolution process.

As a use case, we applied CDE to engineer enzyme longevity. Directed evolution has rarely been used to attempt extension of working lifetime of enzymes *in vivo*, i.e. to increase their catalytic-cycles-till-replacement (CCR) value [20, 21]. This unitless number describes how many reactions an enzyme can catalyse in its *in vivo* lifetime, and CCR values in Arabidopsis vary from <10^2^ to >10^6^ [21]. Histidinol dehydrogenase (CCR = 33 000) has recently been engineered to extend its lifespan [22]. Another short-lived enzyme is cobalamin-independent methionine synthase from *Arabidopsis thaliana* (AtMS1 and AtMS2), which catalyse the generation of methionine (Met) from homocysteine and has a CCR that is ~100-fold below the Arabidopsis median value of 4 × 10^5^. At least a part of the short-lived nature of AtMS may arise from its reactive homocysteine substrate and folate product that put it at risk of self-inactivation [21, 23]. Low CCRs may be associated with damage from catalytic misfires or spontaneous reactions between substrates or products and vulnerable residues in the active site region [21, 24]. Such self-inactivation might be reduced by replacing vulnerable residues [24, 25].

Throughout this study, we outline the stages of a CDE campaign where proteomics can be applied. We employed both targeted and untargeted proteomic approaches to monitor AtMS1 and AtMS2 during CDE. By measuring protein abundance and identifying trade-offs and cellular adaptations in the host, our study demonstrates how mass spectrometry-based proteomics can both identify flaws in population construction and provide insights into directed enzyme evolution within living systems. These results highlight the potential of proteomic strategies as powerful tools for troubleshooting and optimizing synthetic biology workflows, including enzyme lifespan extension.

## Materials and methods

### Plasmid construction

Gene sequences are given in Table S1. Primer sequences used for amplification and sequencing were purchased from Eurofins Genomics (Louisville, KY) and are listed in Table S2. Non-recoded cDNAs of *Arabidopsis* AtMS1 (AT5G17920) and AtMS2 (AT3G03780) were obtained from the Arabidopsis Biological Resource Center (https://abrc.osu.edu/), G13034 and C104738. *MET6* was amplified from genomic DNA of *Saccharomyces cerevisiae* strain BY4742. ORF of AtMS1, AtMS2 and MET6 were cloned into plasmids ArEc-TDH3 and GR-306MP as previously described [26]. Enzymes for polymerase chain reactions (PCR) and cloning were obtained from Thermo Fisher Scientific (Miami, FL).

### Yeast strain construction and media

Yeast strain BY4742 *met6*Δ (MATα; his3Δ1; leu2Δ0; lys2Δ0; ura3Δ0; YER091c::kanMX4) was obtained from Euroscarf (Oberursel, Germany) and grown in YPD medium as previously described [26]. GA-Y319 was cultivated as described previously [26]. Plasmid ArEc-TDH3 containing AtMS1, AtMS2, or MET6 was transformed into BY4742 *met6*Δ using the lithium acetate (LiAc) method with minor modifications [27]. A 5-ml YPD preculture was inoculated with BY4742 *met6Δ* and grown to saturation at 30°C. Five millilitres of YPD per transformation was inoculated with the preculture to set an OD_600_ of 0.3 and grown to an OD_600_ of 0.6. Cells were pelleted by centrifugation (5 min, 1500 g), washed with 5 ml of Milli-Q water per 5 ml of culture, recentrifuged, washed with 150 μl of TE/LiAc (10 mM Tris–HCl, 1 mM Na_2_EDTA, pH 7.5, 100 mM LiAc), and then mixed with 50 μl of TE/LiAc per 5 ml of culture. Ten millilitres of 11 mg/ml of single-stranded salmon sperm DNA were mixed with 300 ng of plasmid DNA and added to the cells. After adding 600 μl of 50% w/v PEG 3350 plus 100 μM of LiAc and 100 μM of TE (10 mM Tris–HCl, 1 mM Na_2_EDTA, pH 7.5), the mix was incubated for 45 min at 30°C with agitation, and afterwards for 20 min at 42°C. Cells were then pelleted by centrifugation (5 min, 700 g) and resuspended in 1 ml of Milli-Q water. Selection was done in synthetic complete (SC) minus histidine for 3 d at 30°C. Selection media were SC-His (6.7 g/l YNB with ammonium sulfate, 1.4 g/l Dropout mix synthetic minus histidine, 20 g/l Bacto Agar (when needed), 2% w/v glucose) for cells containing ArEc-TDH3 plasmids, SC-Leu (6.7 g/L YNB with ammonium sulfate, 1.4 g/l Dropout mix synthetic minus leucine, 20 g/L Bacto Agar (when needed), 2% w/v glucose) for GA-Y319 containing p1_MS, and SC-His-Trp-Leu-Met-Cys (6.7 g/l YNB with ammonium sulfate, 1.4 g/l Dropout mix synthetic minus histidine, tryptophan, leucine, Met and cysteine, 20 g/l Bacto Agar (when needed), 2% w/v glucose) for BY4742 *met6*Δ containing p1_MS. Strains designed for validation of mutant variants were grown in SC (6.7 g/l YNB with ammonium sulfate, 1.4 g/l Dropout mix synthetic minus histidine, tryptophan, uracil, Met, and cysteine, 20 g/l Bacto Agar (when needed), 2% w/v glucose). Transformation of GA-Y319 was done with ScaI-digested GR-306MP harbouring AtMS1 or AtMS2 as previously described [28]. Single colonies were selected for total deoxyribonucleic acid (DNA) isolation and sequencing to confirm integration of the MS genes and the leucine selection marker in the p1 plasmid (1). Primers used are listed in Table S2. Sequence-verified clones were used as the donor strain for protoplast fusion and BY4742 *met6*Δ as recipient. Protoplast fusions were made as before (1). Single colonies were selected for total DNA isolation and sequencing to confirm integration of the MS mutant variants and the uracil selection marker in the p1 plasmid (1). Primers used are listed in Table S2.

### Complementation and growth assays

For complementation assays, single colonies of BY4742 *met6*Δ transformed with ArEc-TDH3 containing AtMS1, AtMS2l or MET6 were grown in 5 ml SC-His at 30°C until saturation. Cells with ArEc-TDH3 only were used as negative control. An aliquot of the cultures was used to inoculate triplicate of 5 ml SC-His at a starting OD_600_ of 0.05. Cultures were grown at 30°C for 5 d, and the OD_600_ was monitored. Growth assays were done similarly but in SC medium minus methionine, cysteine, histidine, tryptophane, and leucine plus 1 mM SeMet.

### Evolution campaigns

Nine independent starting populations expressing AtMS1 or AtMS2 were grown for 29–35 serial passages on Met-free medium containing SeMet. As growth improved, populations were typically split into two or more subpopulations to guard against accidental loss and to enable different evolutionary trajectories from that point forward [1]. Three-millilitres of starter populations were set at OD_600_ = 0.05 in SC minus methionine, cysteine, histidine, tryptophane, and leucine, and cultivated in Corning deep 24-well plates (Millipore Sigma, catalogue no. AXYPDW10ML24C). Plates were covered with Breathe-Easier Sealing Film, Diversified Biotech (Dedham, Massachusetts). Cultivation was performed at 30°C with humidification at 80% and agitation at 800 rpm in an Infors Multitron shaker (Sulzemoos, Germany). SeMet was added from the start at a concentration of 5 μM. Populations were subcultured into fresh medium when they reached saturation. SeMet concentration in the medium was ramped up whenever the evolving populations reached saturation after two days of cultivation. Evolving populations were split at passages 6, 13, and 29. Bulk cultures were analysed for mutations by sequencing PCR amplicons containing the MS ORF and promoter.

### DNA isolation and sequencing

Five-millilitre cultures of evolved populations were used for total DNA extraction as previously described or as follows (1). Cells from a 100 μl aliquot of cultures were mixed with 100 μl of 200 mM lithium acetate plus 1% SDS, and incubated for 10 min at 70°C. 300 μl of ethanol was added, mixed, and centrifuged for 3 min at 15 000 × g. The pellet was washed with 500 μl of 70% ethanol and centrifuged again. After drying for 5 min at 50°C, the pellet was resuspended in 100 μl of milliQ water and centrifuged again for 15 sec. 90 μl were then transferred to a new tube and used as template for PCR amplification and subsequent Sanger sequencing. Primers used are listed in Table S2.

### Analysis of free amino acid pools

Precultures of unevolved and evolved populations were grown in 3 ml SC minus histidine, tryptophane, and leucine, and cultivated until saturation in Corning deep 24-well plates as described above. Aliquots of precultures were inoculated into 3 ml SC minus methionine, cysteine, histidine, tryptophane, and leucine at an OD_600_ of 0.05, and cultivated as before until they reached late log phase. Per culture, a liquid amount, which equals around 300 Mio. cells, was harvested by centrifugation, and the cells were stored at −80°C until extraction. Free amino acids were extracted from the yeast cultures as described previously with the following modifications [29]. 500 μl of 12:5:1 methanol-chloroform-water was added to the frozen cells and mixed thoroughly. Three separate samples were used to determine the spike recovery. They were split into two each, and one aliquot per sample was mixed with 50 μl of a 2.5 mM Met solution. The downstream procedure was as for all other samples, but with half of the used volumes. The samples were incubated for 5 min at 50°C, mixed thoroughly again, and centrifuged for 1 min at 21 000 × g. The supernatant was transferred to a new tube, and the remaining cells were extracted again. 250 μl of chloroform and 375 μl of Milli-Q water were added to the pooled supernatants and mixed by inverting the tubes. The mix was centrifuged for 5 min at 21 000 × g, and 1 ml of the aqueous upper phase was recovered and dried in a speed vac. Derivatization of amino acids was done as described previously with minor changes (4). The dried extracts were resolved in 100 μl of Coupling solution (10:5:2:3 Acetonitrile: Pyridine: Triethylamine: H_2_O), dried again, resolved in 100 μl of Coupling solution, and mixed with 5 μl PITC (Edman’s Reagent; phenylisothiocyanate) (Thermo Fisher Scientific). The solution was then incubated for 5 min at room temperature, dried, and the extract was finally resolved in 125 μl Sample buffer (0.071 g anhydrous sodium dihydrogen phosphate in 100 ml of Milli-Q water, adjusted pH to 7.4, mixed with 5.26 ml acetonitrile). Samples were centrifuged before high-performance liquid chromatography (HPLC) analysis. Reverse-phase HPLC analysis of the free Met pool was carried out as described [30] using a Waters™ (Milford, MA) 2 695 Separations Module coupled to a Waters™ 2 998 PDA Detector. Chromatographic separation was performed on a Supelco™ Discovery® C18 column (Merck KGaA, Darmstadt, Germany) at 25°C. The injection volume was 5 μl. The flow rate was 1 ml/min, and the gradient was as follows. 10 min at 100% eluent A (131 mM sodium acetate, 3.38 mM triethylamine, adjusted to pH 5.7 with acetic acid, v/v 0.06% acetonitrile, 2.4 μM EDTA), 30 s at 46% eluent B (3:2 acetonitrile:water), 2 min at 100% B, 8 min at 100% A. Detection was done at a wavelength of 254 nm. Met identification was done based on comparison with an authentic standard (Merck KGaA, Darmstadt, Germany), and concentrations were calculated based on a standard curve and spike recovery rates.

### Protein expression in *Escherichia coli* and purification

For spike-in standard, *A. thaliana* AtMS1 and AtMS2 genes were synthesized with codon-optimization for expression in *E. coli* and then cloned into pET-15b vector at the NdeI and XhoI restriction sites. The pET-15b-AtMS1 and pET-15b-AtMS2 constructs were expressed in *E. coli BL21(DE3*) using standard protocols as previously described [31]. Briefly, cells were cultured at 37°C with shaking in LB medium containing 100 μg/ml ampicillin and supplemented with 0.5 mM ZnSO_4_ until the OD_600_ of the culture reached 0.6. Protein overexpression was induced by adding filter-sterilized isopropyl β-D-1-thiogalactopyranoside (IPTG) to a final concentration of 0.2 mM for AtMS1 and 0.4 mM for AtMS2. The culture was cooled, and cells were grown with shaking at 18°C overnight and then treated with 100 μg/ml of chloramphenicol 1 hour prior to harvesting to help recover soluble protein from inclusion bodies [32]. Cells were collected *via* centrifugation at 4°C and stored at −80°C.

Harvested cells were resuspended in lysis buffer (300 mM sodium chloride, 50 mM HEPES, pH 7.4). The cells were then incubated with 1 mg/ml lysozyme at 4°C for 1 h, and then lysis was completed *via* sonication. The crude lysate was centrifuged (20 min, 30 000 × *g*, 4 °C), and the pellet was discarded. The clarified supernatant was mixed with Ni^2+^-NTA resin (Millipore Sigma, cat. no. P6611) and incubated at 4°C on a nutator for 30 min. The slurry was used to pour a column, and AtMS1/2 was eluted using elution buffer (300 mM sodium chloride, 50 mM HEPES, pH 7.4, and 250 mM imidazole, pH 7.0). The eluted MS proteins were incubated with thrombin (MP Biomedicals, cat. no. 154163) at a ratio of 1.5 U/mg of protein and then dialyzed against 1 l of 50 mM ammonium bicarbonate at 4°C overnight. The protein was passed through a Ni^2+^-NTA column to remove any protein that still retained the hexahistidine tag, followed by benzamidine-sepharose resin (Cytiva, cat. no. 17512310) to remove thrombin. Protein purity was determined using Coomassie-stained SDS-PAGE. The purified MS proteins were concentrated to 17 mg/ml as determined by UV–visible spectrophotometry using extinction coefficients at 280 nm (ε_280_) calculated by ProtParam (Expasy) [33] and then flash-frozen in 50–100 μl aliquots in liquid nitrogen for storage at −80°C. These were used to verify peptides for targeted MS analysis and as spike-in standards to allow absolute quantification.

### Development of AtMS1 and AtMS2 standards for targeted proteomic analysis

To enable absolute quantification of Arabidopsis Met synthase in yeast lysates, purified AtMS1 and AtMS2 proteins were used as spike-in standards. They were expressed in *E. coli* and purified as described above. The identity and purity of the recombinant proteins were confirmed *via* SDS-PAGE and mass spectrometry. Known concentrations of recombinant AtMS1 and AtMS2 were spiked into *S. cerevisiae* BY4742 lysates that did not contain the construct expressing AtMS1 and AtMS2 for CDE. To ensure specificity, peptides unique to AtMS1 and AtMS2, distinct from the yeast MET6 protein, were selected based on *in silico* digestion and peptide uniqueness analysis. These peptides were used for downstream MRM assay development and quantification. A series of spike-in concentrations was used to construct a standard curve by correlating MS signal intensities with known amounts of AtMS1 and AtMS2. The standard curves enabled absolute quantification of the target proteins in experimental samples by comparing the MRM signal intensities of expressed AtMS1 and AtMS2 peptides to those of the spike-in standards.

### Cultivation and harvest of evolution populations for proteomics analysis

Evolved yeast populations and their corresponding starter populations were grown in selective medium in Corning deep 24-well plates as described above. Per population, 12 cultures á 3 ml were harvested at an OD_600_ between 2.5 and 3.7 and pooled. The cells were harvested by centrifugation at 4°C and washed with 15 ml ice-cold MilliQ water. The final pellet was resuspended in 1 ml ice-cold milliQ water, flash-frozen in liquid nitrogen, and freeze-dried using a Labconco™ FreeZone™ 2.5 l−50°C Benchtop Freeze Dryer (Catalogue no. 710201000).

### Sample preparation for mass spectrometry analysis

Proteins of interest, Met synthases AtMS1 and AtMS2, were analysed from purified proteins and dried yeast extracts, including 84 protein samples derived from unevolved and evolved populations.

Protein extracts were prepared by incubating 10 mg dried yeast samples in 200 μl SDS extraction buffer (7% SDS, 0.1 M Tris, 5 mM TCEP, 1x protease inhibitor, pH 7.5), followed by 10 min incubation at 95°C and 1000 rpm. Samples were transferred for a 1-min treatment in a sonicator water bath to enhance lysis. The samples were centrifuged for 10 min at max speed (~18 000 g), and the supernatant was transferred into a 96-well plate for further processing.

### Protein quantification and digestion

Protein quantification was performed using a bicinchoninic acid (BCA) assay to estimate protein concentration. Proteins were digested using an SP3-aided protocol [34], employing trypsin at a 1:50 enzyme-to-substrate ratio. Digestion was carried out at 37°C for 18 h to ensure complete cleavage. From each sample, 10 μl of the prepared solution was injected for analysis.

### Mass spectrometry analysis

For untargeted analysis, protein samples were injected into an online nanoflow system (0.4 μl/min) using a capillary column (Picofrit, 50 μm tip opening, 75 μm diameter, New Objective, ICT36015030F-50) packed in-house with 15 cm of C18 silica material (3 μm, Dr. Maisch GmbH). This was connected to a Thermo Fusion mass spectrometer in-line with a Dionex Ultimate 3000 series UHPLC. The spray voltage was set to 2 kV, and the heated capillary temperature was maintained at 275°C. For data-dependent acquisition (DDA) experiments, the full MS resolution was set to 60 000 at *m/z* 200, with an AGC target of 300% and auto injection time. The mass range was set from 350 to 1500 *m/z*. The AGC target value for fragment spectra was set to ‘standard’ with a resolution of 15 000 and auto-injection time. An intensity threshold of 5 × 10^4^ was applied, and the isolation width was set at 1.6 *m/z*. Normalized collision energy was set to 30%. The column flow rate was 0.4 ul·min-1 with the following elution profile: 0–0.5 min 2% (v/v) ACN, 0.1% (v/v) FA to 6% (v/v) ACN, 0.1% (v/v) FA; 0.5–25 min 6% (v/v) ACN, 0.1% (v/v) FA to 27% (v/v) ACN, 0.1% (v/v) FA; 25–26 min 27% (v/v) ACN, 0.1% (v/v]) FA to 35% (v/v) ACN, 0.1% (v/v) FA; 26–26.1 min 35% (v/v) ACN, 0.1% (v/v) FA to 80% (v/v) ACN, 0.1% (v/v) FA; 26.1–26.5 min 80% (v/v) ACN, 0.1% (v/v) FA, 26.5–26.7 min 80% (v/v) ACN, 0.1% (v/v) FA to 2% (v/v) ACN, 0.1% (v/v) FA; 26.7–30 min 2% (v/v) ACN, 0.1% (v/v) FA.

Raw files were processed using the MaxQuant software (Version 2.4.0.0, [35]) using default settings. Trypsin was used as protease and up to two allowed missed cleavages. Cysteine carbamidomethylation was set up as a fixed modification, and oxidation of Met and N-terminal acetylation as a variable modification. Peptide identification required a minimum of seven peptides, and a maximum of five modifications was allowed. Match-between-run function was enabled. Global data normalization was done using the MaxQuant label-free quantitation (LFQ) algorithm with LFQ minimum ratio count set to two and fast LFQ enabled. The following fasta files were used: sequences for the evolved proteins, plus the reference fasta file UP000002311 for *S. cerevisiae* was downloaded from Uniprot. MaxQuant output files were used to further analyse and visualize in R.

For targeted analysis by multiple reaction monitoring, digested peptides were loaded onto an Aeris 3.6 μm peptide XB-C18 100 Å, LC column 50 × 2.1 mm (Phenomenex) using a Thermo UltiMate 3000 RSLCnano System coupled to an Thermo TSQ Altis Triple Quadrupole MS. The column was heated to 55°C and the column flow rate was 0.4 ml·min^−1^ with the following elution profile: 0–20.5 min 2% (v/v) acetonitrile, 0.1% (v/v) formic acid to 22% (v/v) acetonitrile, 0.1% (v/v) formic acid; 20.5–27.5 min 22% (v/v) acetonitrile, 0.1% (v/v]) formic acid to 35% (v/v) acetonitrile, 0.1% (v/v) formic acid; 27.5–28.5 min 35% (v/v) acetonitrile, 0.1% (v/v) formic acid to 97% (v/v) acetonitrile, 0.1% (v/v) formic acid; 28.5–29.2 min 97% (v/v) acetonitrile, 0.1% (v/v) formic acid, 29.2–29.5 min 97% (v/v) acetonitrile, 0.1% (v/v) formic acid to 2% (v/v) acetonitrile, 0.1% (v/v) formic acid; 29.5–35 min 2% (v/v) acetonitrile, 0.1% (v/v) formic acid. The list of peptide transitions used for selected reaction monitoring (SRM-MS) is provided in Table S3. Peak area of ions for targeted peptides was determined using the Skyline software package version 24.1.0.199 (reference for Skyline: https://pubmed.ncbi.nlm.nih.gov/20147306/). Purified AtMS1 and AtMS2 proteins, which were expressed and extracted from *E. coli*, were used as internal standards. The protein standards were spiked into yeast lysates at known concentrations to generate a standard curve as described above. Absolute protein concentrations in the yeast samples were subsequently determined by comparing the MS signal intensities of recombinant proteins to those of the spiked-in standards. Further data analysis was performed using R.

The mass spectrometry proteomics data have been deposited to the ProteomeXchange Consortium *via* the PRIDE partner repository with the dataset identifier PXD069101 and 10.6019/PXD06910 [36].

## Results and discussion

### Host selection and validation for directed evolution

The selection of a platform host depends on the target gene’s origin and the destination organism, considering subcellular localization of the target protein, posttranslational modifications (PTM), intracellular pH, redox conditions, and oxygen levels. PCR and phenotypic tests (e.g. growth assays in defined media) are common techniques to validate the chosen background strain (e.g. a knockout mutant in *S. cerevisiae*). However, complementing these analyses with targeted proteomics can confirm strain suitability and verify the presence and absence of proteins (Fig. 1A). In addition, untargeted proteomics can reveal cellular changes that may influence selection during the evolution experiment.

We selected *S. cerevisiae* as the evolution host due to its ‘plant-like’ cellular conditions, including compartmentalization, metabolite and cofactor availability, and shared metabolic pathways. For our approach, the AtMS1 and AtMS2 genes were codon-optimized for yeast and expressed from the plasmid ArEc-TDH3 in a *met6*Δ knockout mutant (e.g. yeast Met synthase deleted) to perform complementation assays in a methionine-deficient (−Met) medium (Fig. S1). Targeted proteomics can support this validation step if the desired phenotype is not achieved, enabling the assessment of (i) protein abundance, (ii) solubility, and (iii) interactions with the cellular machinery.

### Generation of evolution starter populations and verification

**Figure 2:**
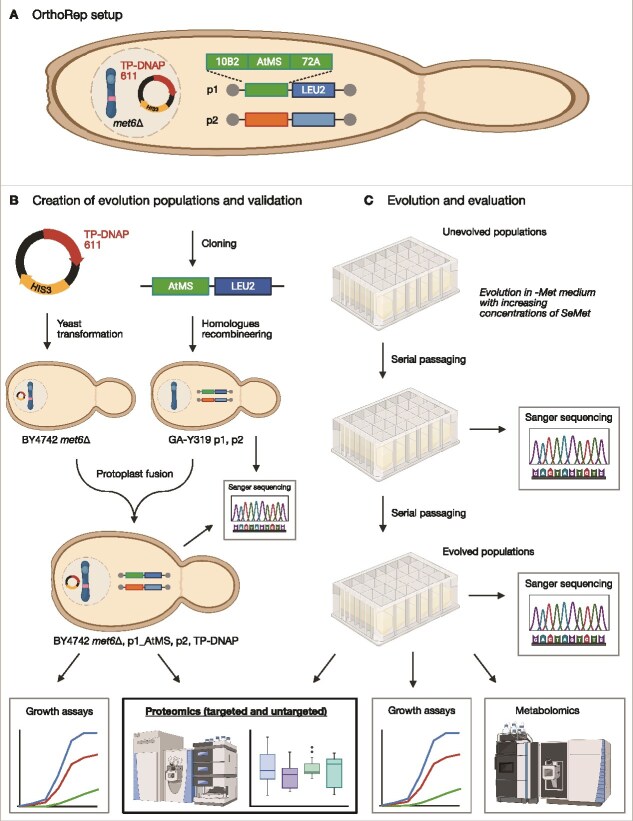
Assembly of OrthoRep cells and workflow of the presented study. A. Evolution populations were created in a BY4742 *met6*Δ yeast background. A nucleus-localized plasmid under control of a HIS3 marker encoded the error-prone polymerase TP-DNAP1 611. p1 encodes the AtMS target gene and a LEU2 selection marker. p2 encodes the RNA-polymerase and a p2-specific DNA-polymerase. B. Evolution starter populations were created and later verified by Sanger sequencing, growth assays, and proteomics (targeted and untargeted). C. Accumulated mutations in target genes in evolved populations were determined by Sanger sequencing, and cellular phenotypes were evaluated by growth assays, metabolomics, and proteomics (targeted and untargeted). The figure was created in BioRender. Bathe, U. (2025) https://BioRender.com/gq2ufl5.

When selecting a CDE system, mutation rate and bias, and overall system design should align with the experiment’s objectives. For this study, we utilized the OrthoRep system due to its ability to achieve an orthogonal replication mutation rate between 10^5^ and 10^6^ times higher than the genomic mutation rate [7, 37]. OrthoRep comprises an orthogonal error-prone polymerase expressed from a nuclear plasmid, and two cytosol-localized linear plasmids (p1 and p2) that facilitate self-replication, encode an RNA-polymerase, a marker gene, and the gene of interest (Fig. 2A). To assemble this system (Fig. 2B), AtMS1 or AtMS2 and the marker gene were first integrated into the p1 plasmid in the strain GA-Y319 [26]. Successful integration was confirmed by estimating the p1 plasmid size on an agarose gel (Fig. S2A) and by verifying the target gene *via* Sanger sequencing. In addition, we recommend that targeted proteomics analysis, as described earlier, be employed here to confirm the target protein’s expression and functionality in the OrthoRep setup.

**Figure 3:**
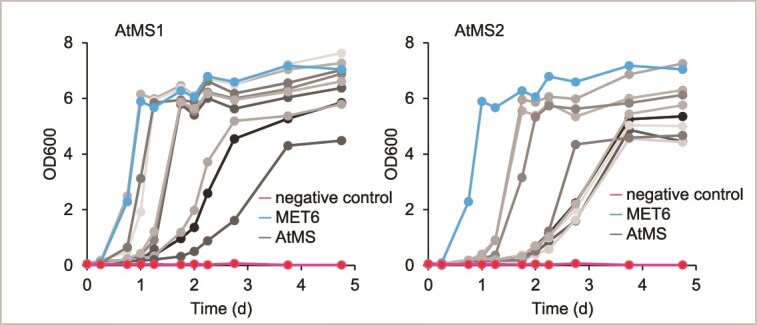
Test of evolution starter populations. Complementation of BY4742 *met6*Δ with AtMS1 and AtMS2 expressed from p1 after protoplast fusion. Strains having the p1 with no AtMS gene served as negative control but were supplemented with Met in the growth medium as positive control.

To transfer the OrthoRep machinery to the chosen platform host, we performed protoplast fusion between the obtained donor strain carrying the p1 plasmid and the *met6*Δ recipient strain with a plasmid expressing the error-prone polymerase (Fig. 2B) [26]. Protoplast fusion clones were verified by gel analysis (Fig. S2B) and Sanger sequencing of the target gene on p1 and complementation assays in −Met medium (Fig. S2B; Fig. 3). Proteomics can be employed here to assess whether the yeast protein machinery, including the CDE system and the protein of interest, shows any unexpected alterations (Fig. 1B; Fig. 2).

**Figure 4:**
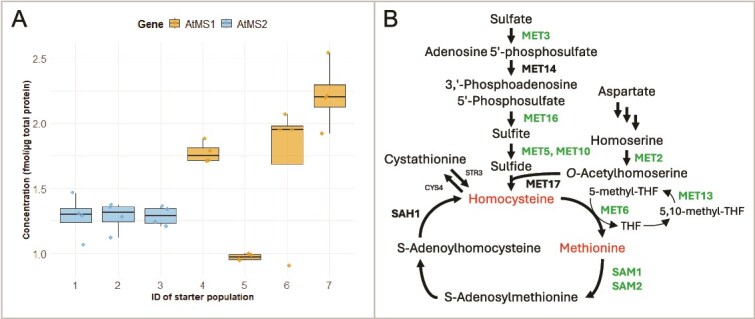
Proteomics of Arabidopsis AtMS enzymes expressed from p1 and MET related enzymes in unevolved yeast populations. A. Targeted protein analysis of Arabidopsis AtMS enzymes in evolution starter populations; *n* = 4. B. Illustration of the Met biosynthesis pathway in yeast. Identified Met-related enzymes are highlighted in green. Additionally, MET18 (part of the cytosolic iron–sulphur protein assembly), MET22 (bisphosphate-3′-nucleotidase), MET8 (involved in the biosynthesis of siroheme), and METW were detected. The figure was modified from [59].

For our study, four AtMS1 and three AtMS2 starter populations were selected, and we first performed targeted proteomics using multiple reaction monitoring mass spectrometry (MRM-MS; Fig. 2B) to confirm the presence and accurately measure the abundance of AtMS1 and AtMS2 based on spike-in standards (Table S3). We confirmed the presence of AtMS1 and AtMS2 proteins, which were expressed between 1 and 2.5 fmol/μg total protein (Fig. 4A). While AtMS2 is uniformly expressed among the three investigated evolution starter populations, AtMS1 expression differs between the populations. It demonstrates that multiple populations should be used to start an evolution campaign; the differences most likely come from different copy numbers of p1 [7].

Untargeted proteomics was then used to investigate cellular changes in the host cells (Fig. 2B). We focused our analysis on Met biosynthesis and related pathways (Fig. 4B; Table S4; Fig. S3) that shows abundance of MET10, MET16, MET18, MET2, MET22, MET3, MET5, MET6, MET8, SAM1, SAM2, and METW in unevolved populations. Interestingly, the overall trend is that Met enzymes are more abundant in populations of AtMS2 compared to AtMS1. This illustrates that expression of foreign enzymes in a CDE system can have different impacts on the host cells’ metabolism and their adaptation, even though such enzymes share high sequence identity, e.g. AtMS1 and AtMS2 proteins are 92.81% identical.

Unexpectedly, we also found that MET6 was detected in the protoplast fusion clones, despite the evolution starter populations being generated from a *met6*Δ knockout strain (Fig. 2B). This likely resulted from chromosomal contamination during the fusion process. Rather than excluding these clones, we continued with them to showcase how proteomic analysis can reveal such unexpected events and add value to a CDE campaign, as detailed below.

### Monitoring ongoing evolution

Running CDE experiments is considered hands-off and low-effort venture. Beneficial mutations in the target gene are selected when the target’s performance is coupled to growth or another readable phenotype. Over time, these mutations accumulate, and the evolution is stopped when a desired phenotype is achieved. In practice, this means serial passaging (Fig. 2C), typically every couple of days, with growth phases in between. To evolve AtMS1 and AtMS2, we used a selection strategy in which the concentration of the toxic Met analogue L-selenomethionine (SeMet) in the medium was gradually raised from 5 μM to 1 mM (Fig. S4). SeMet toxicity is believed to be attributed to the depletion of reduced thiol compounds [38]. The toxic effect can be outcompeted by supplying Met or cysteine [38–40]. Therefore, we hypothesize that this strategy can favour increased Met synthase-driven flux to Met, which expands the free Met pool and so competes out the analogue’s toxicity. A similar approach has been applied to other metabolites in directed evolution before [22, 41]. Although the starting populations contained MET6, therefore were not exclusively dependent on AtMS1 and AtMS2 expressed from p1, we argue that improved AtMS1 and AtMS2 can support flux towards Met production above and beyond the endogenous MET6 capability and therefore, can contribute to the anticipated goal of outcompeting the toxic effect of SeMet.

Starting populations expressing AtMS1 or AtMS2 were grown for 29–35 serial passages on Met-free medium containing SeMet. During the campaigns, it is recommended to collect sequencing data of the target gene to verify that mutations accumulate (Fig. 2C). Such information may also help to follow the evolutionary trajectory. While such analysis is limited to sequence information, proteomics is a complementary tool to monitor the actual phenotype (Fig. 1C). For example, targeted proteomics can be used to assess changes in the abundance of proteins of interest. Additionally, untargeted proteomics can help identify global cellular responses to the selection regime, revealing potential off-target effects (e.g. genomic mutations) or adaptive mechanisms in the host (e.g. epigenetic adaptation). Such data can provide critical feedback to guide adjustments in experimental conditions and ensure that the desired phenotypic traits are being optimized effectively.

### Validation of evolved phenotypes

**Figure 5:**
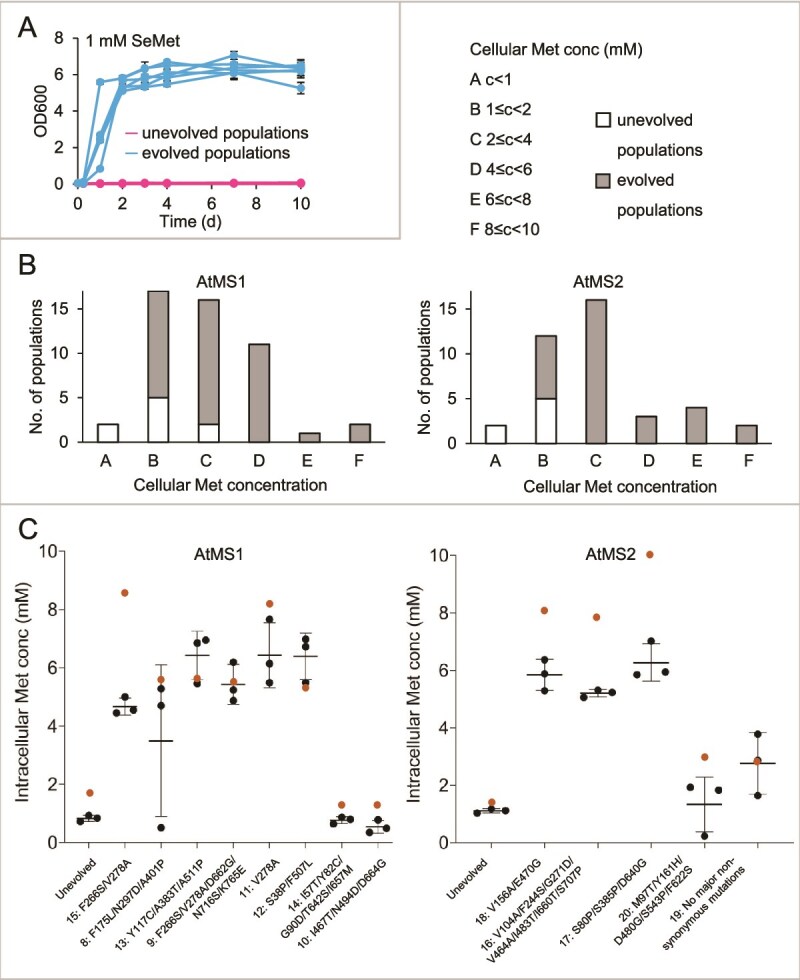
Analysis of evolved AtMS populations. A. Growth comparison of evolved and unevolved populations in presence of 1 mM SeMet. B. Free intracellular Met pools of all unevolved and evolved populations; *n* = 1. C. Evolved populations with the highest Met content from B (orange dots) were subjected again to free Met pool measurements; *n* = 3. Representative unevolved populations are given for comparison.

Once a desired phenotype is achieved, the evolution is stopped, and evolved sequences are verified (Fig. 2C). We halted the AtMS campaigns at a final SeMet concentration of 1 mM as complex secondary toxic effects are more likely at higher concentrations [38, 42]. In total, 72 populations were obtained. Figure 5A shows the growth of six representative evolved populations compared to the corresponding unevolved populations in 1 mM SeMet. To verify increased intracellular Met concentrations, we screened the 72 evolved populations in single replicates (Fig. 5B). Cellular free Met levels of 1–2 mM in most unevolved populations and a marked upshift in evolved populations towards levels as high as 10 mM were observed, proving that SeMet treatment can favour increased Met synthase-driven flux to Met. Therefore, we chose eight and six populations with AtMS1 and AtMS2, respectively, to measure free Met pools in triplicate to gain statistical confidence about our data (Fig. 5C). Consistent with our previous screening, we confirmed high Met-producing and low Met-producing populations. Free Met pools may differ slightly from the first evaluation of populations due to the timepoint of harvest.

To determine what changes accumulated in CDE, the mutated sequences were isolated from the populations and subjected to sequencing. The chosen AtMS populations from the Met pool measurements were used to detect mutations (Fig. S5). Mutant AtMS variants were recovered having a mix of nonsynonymous, synonymous, and promoter mutations. Most of them replaced the wildtype base in all gene copies of the population, proving selection was applied to AtMS1 and AtMS2 on p1.

**Figure 6:**
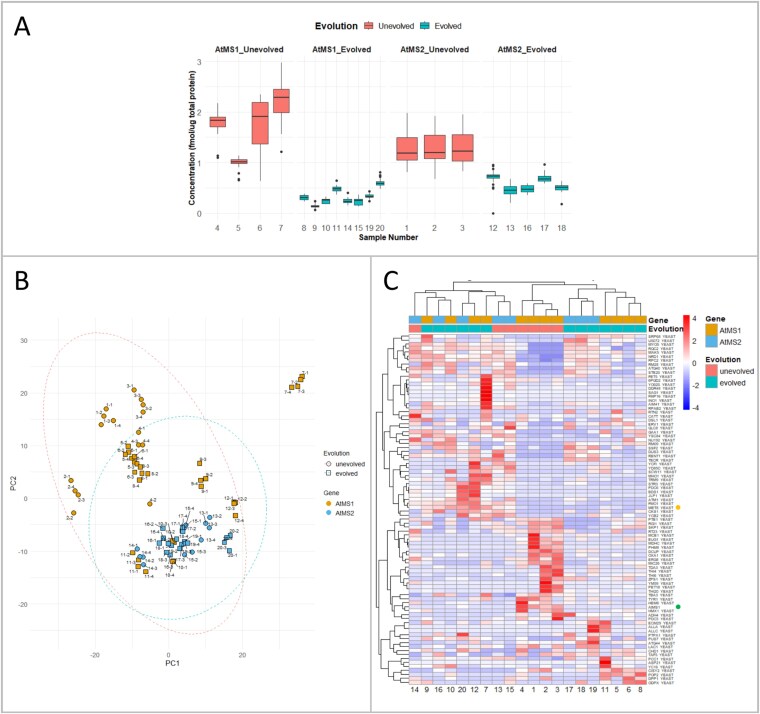
Protein analysis of AtMS1 and AtMS2 in evolved and unevolved yeast populations. A. Concentrations of AtMS1 and AtMS2 proteins in evolved and unevolved yeast populations (labelled 1–20), quantified by mass spectrometry; *n* = 4. B. Untargeted proteomics analysis of both evolved and unevolved populations expressing AtMS1 or AtMS2. Left, PCA plot illustrates clustering of AtMS1 and AtMS2 samples as well as evolved and unevolved populations. Right, heatmap illustrating row-scaled abundances of significantly changing proteins between populations (log2 FC > 2 and *P* < 0.05 determined by *t*-test). AtMS1 (green dot) and MET8 (yellow dot) were among the proteins most significantly changing in abundance based on selected thresholds.

As outlined above, proteomics can be applied to validate evolved phenotypes by directly assessing changes in abundance of proteins as outcomes of the evolution process in a targeted and untargeted manner (Fig. 1C; Fig. 2). With that, broader cellular adaptations can be identified that might accompany the evolved phenotype. In our study, we hypothesized that longer AtMS enzyme lifetime (i.e. higher protein stability) would correlate with higher protein abundance in evolved populations. To further assess protein longevity, approaches such as pulse-chase SILAC and 15^N^-labelling could be employed to directly measure turnover rates of AtMS1 and AtMS2 [43–45]. As an initial test for abundance, we used targeted proteomics to measure AtMS in evolved populations compared to unevolved ones (Fig. 6A). Lower AtMS protein levels were detected, indicating that accumulated major mutations did not correlate with evolved cellular phenotypes, e.g. elevated free Met pools and improved growth in medium with SeMet. While improved enzyme kinetics can, in principle, be the reason for increased Met flux at lower protein abundance, such improvements of central metabolic enzymes are likely modest [46, 47]. Instead, the presence of MET6 caused relaxed selection on AtMS1 and AtMS2. As protein expression from p1 can be considered a metabolic burden to the cell, expression might be reduced if the protein is not required for survival. Therefore, we think that lower AtMS1 and AtMS2 expressions in our settings could have resulted in a metabolic fitness benefit. If the evolution campaign had continued, AtMS expression might have been completely switched off, resembling natural gene loss as a process of adaptation [48].

We then used an untargeted proteomics approach to investigate broader host strain adaptation and investigated any changes specifically in the Met biosynthesis and related pathways of evolved populations (Table S4; Fig. S6; Fig. 6B and C). Principal component analysis revealed partial separation of evolved and unevolved populations, with AtMS1 and AtMS2 samples clustering in distinct regions (Fig. 6B). While evolved and unevolved populations showed some divergence along PC1 and PC2, there was also substantial overlap, particularly within AtMS2 samples. This indicates that although evolutionary pressure led to measurable adaptation in proteomic profiles, the extent of divergence was only modest. Notably, AtMS1 samples displayed greater dispersion, whereas AtMS2 samples clustered more tightly.

In total, 88 proteins showed significant differences in abundance across evolution and AtMS groups with a *P* value < 0.05 and log2 fold change >2. The heatmap of these proteins reveals distinct patterns separating evolved from unevolved populations (Fig. 6C; Table S5). These findings indicate a remodelling of protein abundance during CDE and suggest that proteomic adaptation involved both shared and AtMS-dependent responses.

When comparing Met-related enzymes, we identified adaptations in abundance that correlate with increased free Met pools, e.g. the abundance of Met biosynthesis enzymes MET3, MET16, MET10/MET5, MET2, and MET6 (along with related Met enzymes MET8 and METW) were increased in AtMS1 populations (Fig. S6). Interestingly, these enzymes were not increased in the AtMS2 populations. We therefore hypothesized that the increased free Met pool in AtMS2 populations came from improvements introduced into AtMS2. The isolated AtMS2 variants were tested in fresh cells, but no SeMet resistance was observed, and the origin of the improved phenotype in AtMS2 populations remains unclear. However, we demonstrated that the applied selection pressure affected the host’s metabolic network and the proteome, and the findings highlight the complementary strengths of targeted and untargeted proteomics for a better understanding of evolved phenotypes and system-wide effects.

Directed evolution-acquired mutations in an enzyme might impede with its *in vivo* regulation, e.g. feedback inhibition, which can cause increased product formation. To our knowledge, there is no feedback inhibition of Met synthase, and we argue that there is likely none because accumulation of its reactive substrate homocysteine is toxic to the cell [49]. Instead, it is more likely that the Met biosynthesis is feedback-regulated earlier in the pathway [50].

In addition, one might consider other possible scenarios that can influence the outcome of an evolution campaign: (i) The copy number of the p1 plasmid can verify per cell and is dependent on the error-prone polymerase used [51], therefore, can account for differences between evolved populations or between unevolved and evolved populations. Here, qPCR can help and was used successfully in the past to verify p1 copy numbers [7, 52]. (ii) Synonymous mutations can accumulate and improve enzyme expression to meet the host cell’s preference for codon usage (even though the target gene has been codon-optimized) [26, 28]. Non-synonymous mutations in evolved sequences should be tested independently of evolved synonymous mutations to verify their contribution to the evolved phenotype. (iii) Promoter mutations may accumulate and improve expression, in particular in OrthoRep, as the promoter is subjected to hypermutation too [53]. (iv) False-positives might arise in an evolved population due to metabolic crosstalk between cells that allows ‘cheaters’ to benefit from truly evolved cells [54, 55].

Proteomics offers an unbiased approach to confirm gene deletions and to detect unexpected protein expressions or compensatory changes during evolution experiments. While adding tags like FLAG or HA are useful for targeted detection in many contexts, our label-free method provides a comprehensive view without requiring genetic modification and avoids the tag itself or its co-evolution affecting results in CDE. Mass spectrometry–based proteomics provides a flexible and unbiased readout for CDE experiments, as it is applicable to virtually any target protein once a reference proteome (or the sequence of a mutated variant) is available [22, 56, 57]. In addition, targeted MS approaches such as MRM allow absolute or near-absolute quantification with high precision and broad dynamic range, surpassing the semi-quantitative nature and antibody dependence of Western blotting. Importantly, proteomics enables simultaneous measurement of the evolving target protein together with its pathway context in the same experiment, ensuring internally consistent comparisons across enzymes and conditions. Our study did not aim for near complete proteome coverage but rather an economical use that could be employed across populations and timepoints in a campaign; the relatively short 26 min gradient and 30 min acquisition time inevitably limited the number of identifications compared with longer gradients uses in deeper yeast proteomics [58]. Future studies employing extended gradients or data-independent acquisition (DIA) could substantially increase proteome depth and completeness if required, offering complementary insights to the high-throughput MS1-based workflow used in our study. Though, the proteomics approach used here still demonstrated high sensitivity and statistical confidence, enabling the detection and quantification of even very low protein abundances, down to femtomoles per microgram of the yeast background proteome.

Previously, mass spectrometry was used in CDE that evolved compact zinc finger degrons to enable selective, potent, and tunable protein degradation. The authors employed peptide mass spectrometry to confirm the absence of off-target protein degradation, an essential step to ensure the specificity of their evolved constructs [16].

### Beyond phenotype validation: additional proteomics approaches

To further enhance the insights gained from CDE, proteomics analyses can be integrated at other stages in the process (Fig. 1D). First, post-translational modification analysis can provide valuable information on how target proteins are regulated or modified in the host or during CDE, potentially revealing novel mechanisms of adaptation (e.g. change in phosphorylation, acetylation, oxidation or glycosylation patterns). Second, interaction proteomics, such as co-immunoprecipitation coupled with mass spectrometry, can be employed to identify changes in protein–protein interaction networks. This is particularly useful for understanding how the evolved phenotype integrates into the cellular machinery. Third, proteomics-based quantification of metabolic enzymes can link phenotypic changes to metabolic shifts, providing a more holistic understanding of how evolution reshapes cellular physiology. These additional analyses can deepen the mechanistic understanding of evolved phenotypes and inform strategies for optimizing the evolutionary process further.

## Conclusion

CDE experiments can greatly benefit from implementing mass spectrometry-based proteomics because it uncovers cellular changes which complements what can be detected on the DNA or RNA levels. As shown in our study, it can give valuable insights into the actual phenotype of the platform cells during a campaign. Using *Arabidopsis* AtMS1 and AtMS2 as an example, we demonstrated at what checkpoints both targeted and untargeted proteomics can be applied, what the delivered value would be, and outlined other potential applications during and after evolution. We discovered the value of proteomics to reveal unexpected flaws in population construction through the retention of some MET6 capacity; information that was important to interpret our campaign strategy based on SeMet-dependent selection. It also revealed how different target proteins subjected to evolution can have varying effects on the host cell, even though they share high sequence similarity. Using systems-level analysis, we further identified changes in protein expression of diverse pathways in yeast as a result of the applied selection. Combined, our results demonstrate the benefits of complementing continuous evolution with proteomics analysis.

## Supplementary Material

Supplementary_Material_ysaf017

Suppl_Table_S4_ysaf017

Suppl_Table_S5_ysaf017

## Data Availability

All data generated in this study are available in the main text, in the figures or in the supplemental information. Data are also available on bioRxiv: https://doi.org/10.1101/2025.03.03.641153. The mass spectrometry proteomics data have been deposited to the ProteomeXchange Consortium *via* the PRIDE partner repository with the dataset identifier PXD069101 and https://www.ebi.ac.uk/pride/archive/projects/PXD069101 [36].

## CRediT statement

Katharina Belt (Conceptualization, Data curation, Formal analysis, Investigation, Methodology, Validation, Visualization, Writing—original draft, Writing—review & editing), David Obe (Investigation, Methodology), Mark A Wilson (Conceptualization, Formal analysis, Funding acquisition, Methodology, Supervision, Writing—original draft, Writing—review & editing), A Harvey Millar (Conceptualization, Formal analysis, Funding acquisition, Supervision, Writing—original draft, Writing—review & editing), Ulschan Bathe (Conceptualization, Data curation, Formal analysis, Funding acquisition, Investigation, Methodology, Project administration, Validation, Visualization, Writing—original draft, Writing—review & editing).

## Material availability statement

All material used in this study is available upon request and signing a MTA.

## Notes

We dedicate this work to the memory of Andrew D. Hanson, whose pioneering contributions and scientific vision continue to inspire the field of synthetic biology.
