# Supplementary material for: Harnessing mass spectrometry-based proteomics for continuous directed evolution
Source: Synth Biol (Oxf). 2025 Dec 4;10(1):ysaf017. doi: 10.1093/synbio/ysaf017 (PMC12765272; doi:10.1093/synbio/ysaf017)
Supplement: Supplementary_Material_ysaf017 [file supplementary_material_ysaf017.pdf]

Supplemental Information

for

Harnessing Mass Spectrometry-Based Proteomics for Continuous Directed Evolution

Katharina Belt [1], David Obe [2], Mark A. Wilson [2], A. Harvey Millar [1], Ulschan Bathe [3]*

[1] ARC Centre of Excellence in Plant Energy Biology, School of Molecular Sciences, University of Western Australia, Crawley 6009 WA, Australia

[2] Department of Biochemistry and Redox Biology Center, University of Nebraska, Lincoln, NE 68588, USA

[3] Biotechnology of Horticultural Crops, TUM School of Life Sciences, Technical University of Munich, Liesel-Beckmann-Str. 1, Freising 85354, Germany

[*] Author for communication: ulschan.bathe@tum.de

SUPPLEMENTAL MATERIAL

Figure S1 Complementation of BY4742 met6Δ with AtMS1 and AtMS2

Figure S2 Analysis of evolution starter populations by gel to verify the size of plasmid p1

Figure S3 Untargeted proteomics analysis of unevolved populations

Figure S4 Evolution strategy using SeMet

Figure S5 Mutations recovered in AtMS sequences after evolution

Figure S6 Proteomics analysis of Met related proteins in evolution populations

Table S1 Sequence list of used genes

Table S2 List of used primers

Table S3 Mass spectrometry data of AtMS1 and AtMS2

Table S4 Untargeted proteomics data of evolved and unevolved yeast strains

Table S5 Comparison of protein abundance between evolved and unevolved populations

SUPPLEMENTAL FIGURES

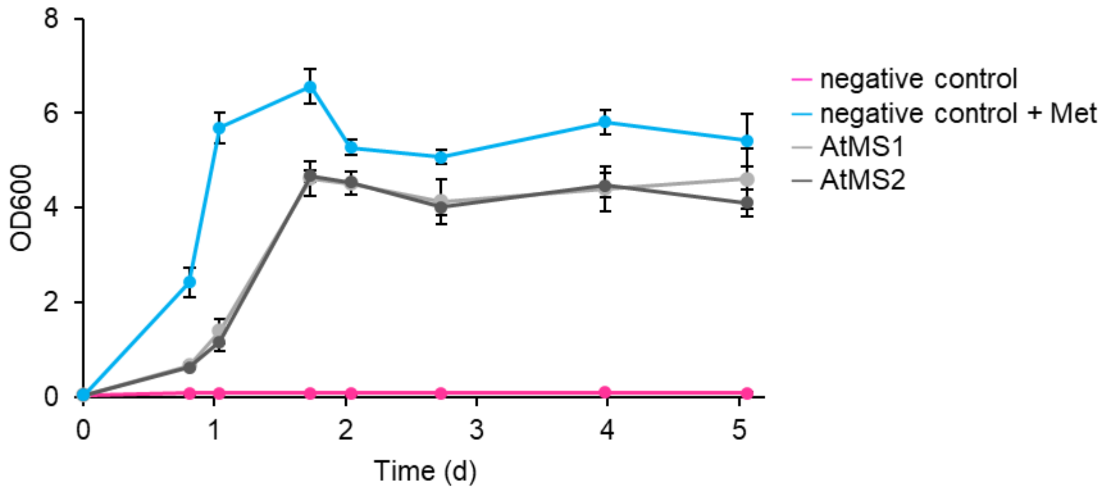

Figure S1: Complementation of BY4742 *met6*Δ with AtMS1 and AtMS2. Proteins AtMS1 or AtMS2 were expressed from the plasmid ArEc-TDH3. The vector alone (i.e., no MS gene) served as negative control (magenta) but was supplemented with Met in the growth medium as positive control (blue).

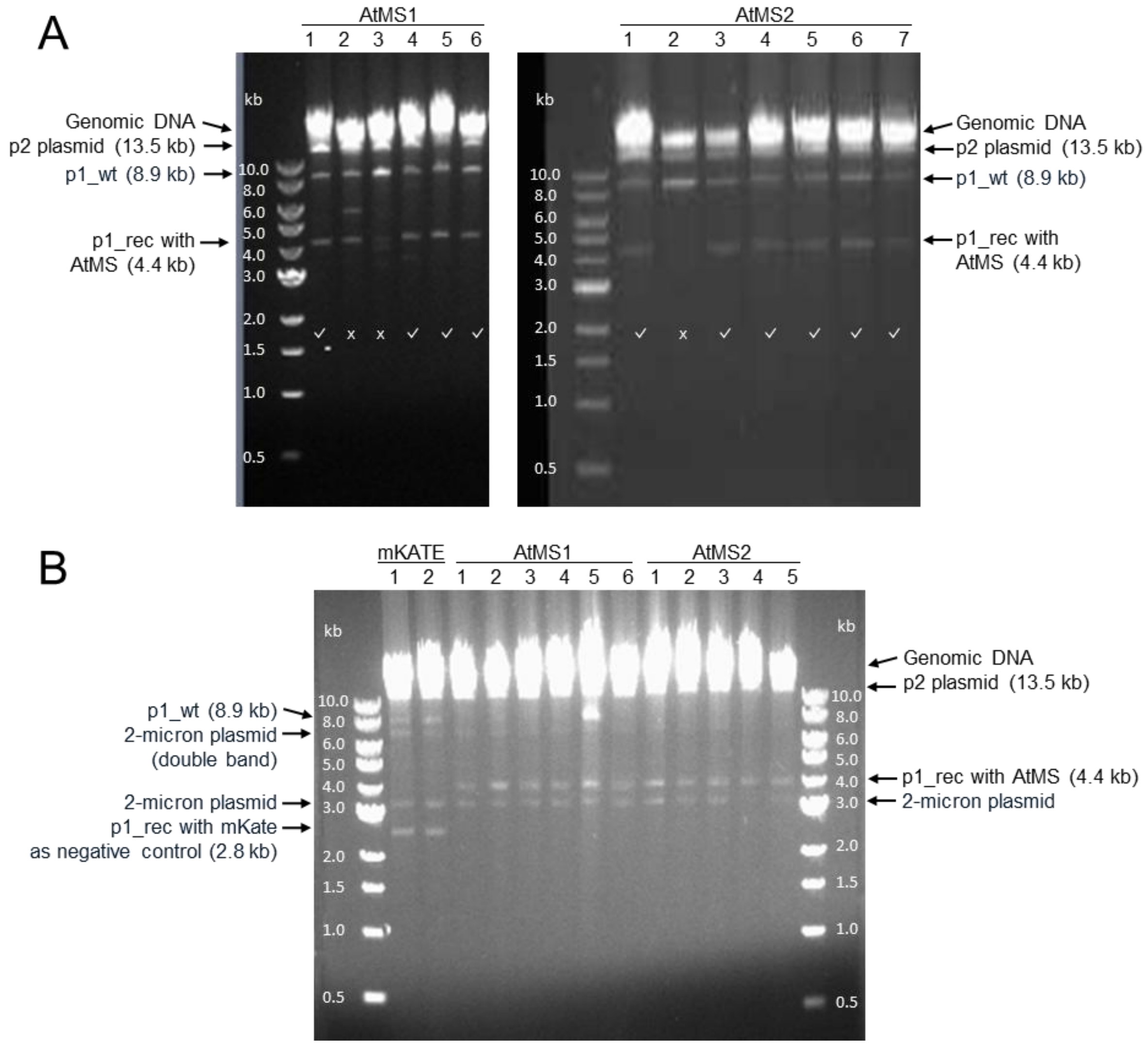

Figure S2. Representative gel analysis of yeast clones with AtMS on the p1 plasmid. Yeast clones were grown in 5 mL of liquid medium from which total DNA was isolated. 1 µg of DNA was then loaded to a 0.8 % agarose gel and ran for 90 min at 80 V. A, The band pattern of GA-Y319 strains with AtMS1 and the selection marker integrated on p1 where gel-analyzed to select clones for protoplast fusion. Positive clones (✓) and negative clones (x) are indicated. B, Protoplast fusion clones were verified by gel Shown are samples from protoplast fusion clones that have either mKATE on the p1 plasmid (negative control), AtMS1 or AtMS2. The 2-micron plasmid is a small multi-copy DNA plasmid and is often found in strains of *Saccharomyces cerevisiae*.

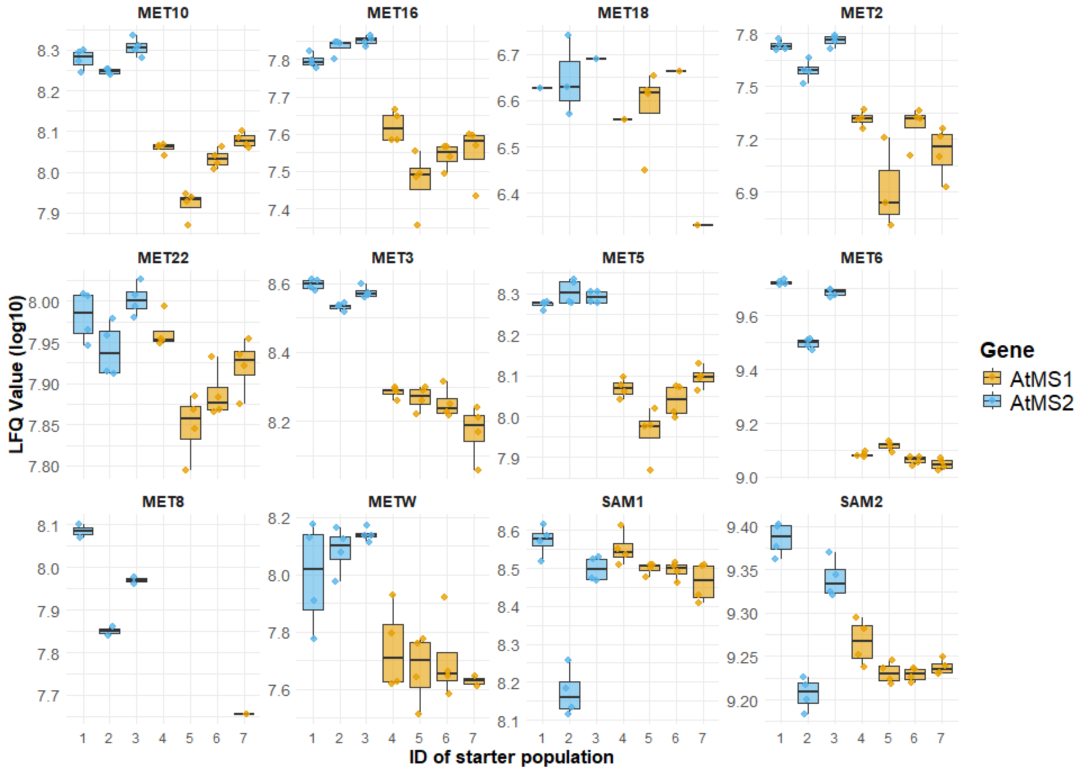

Figure S3: Untargeted proteomics analysis of unevolved populations. Cells express AtMS1 or AtMS2 from p1. Expression of Met synthase-related proteins with corresponding LFQ values obtained from mass spectrometry are shown; n=4.

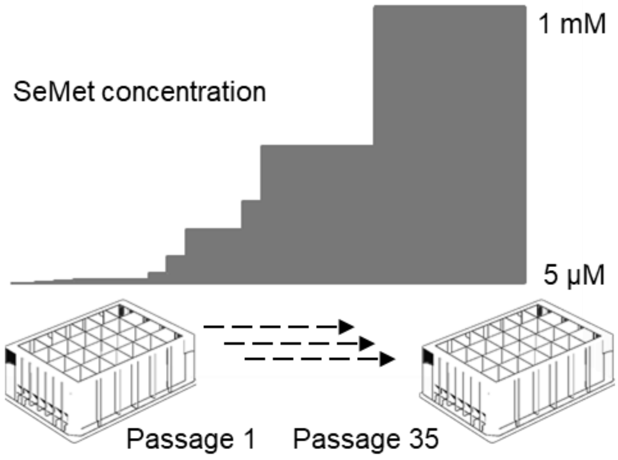

Figure S4: SeMet treatment to evolve AtMS. AtMS evolution campaigns were run for 29–35 passages, and SeMet concentration was gradually raised from 5 µM to 1 mM.

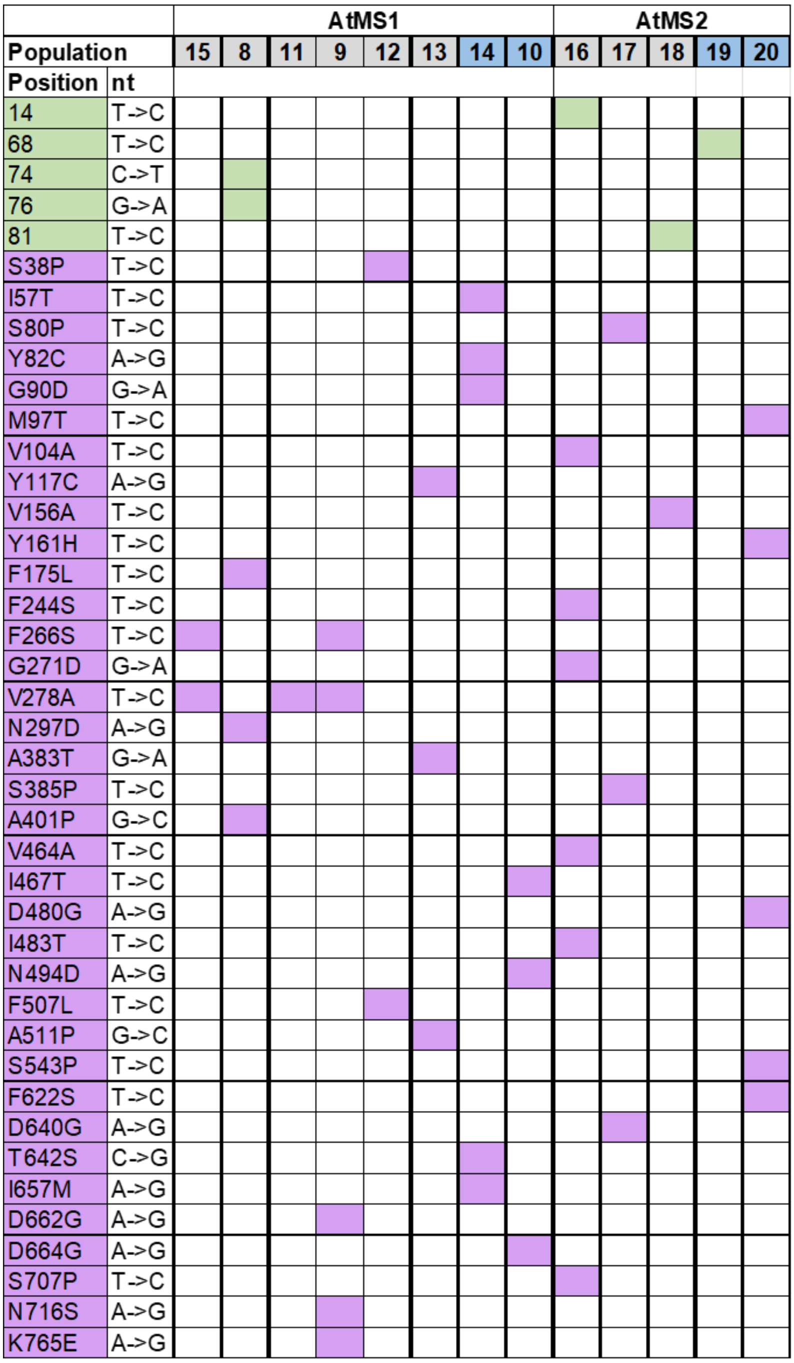

Figure S5. Major mutations recovered in evolved AtMS populations. Populations with high free Met pools chosen for further analysis (grey) accumulated promoter (green) and major ORF nonsynonymous (purple) mutations. Representative populations with low free Met pools (blue) are given for comparison.

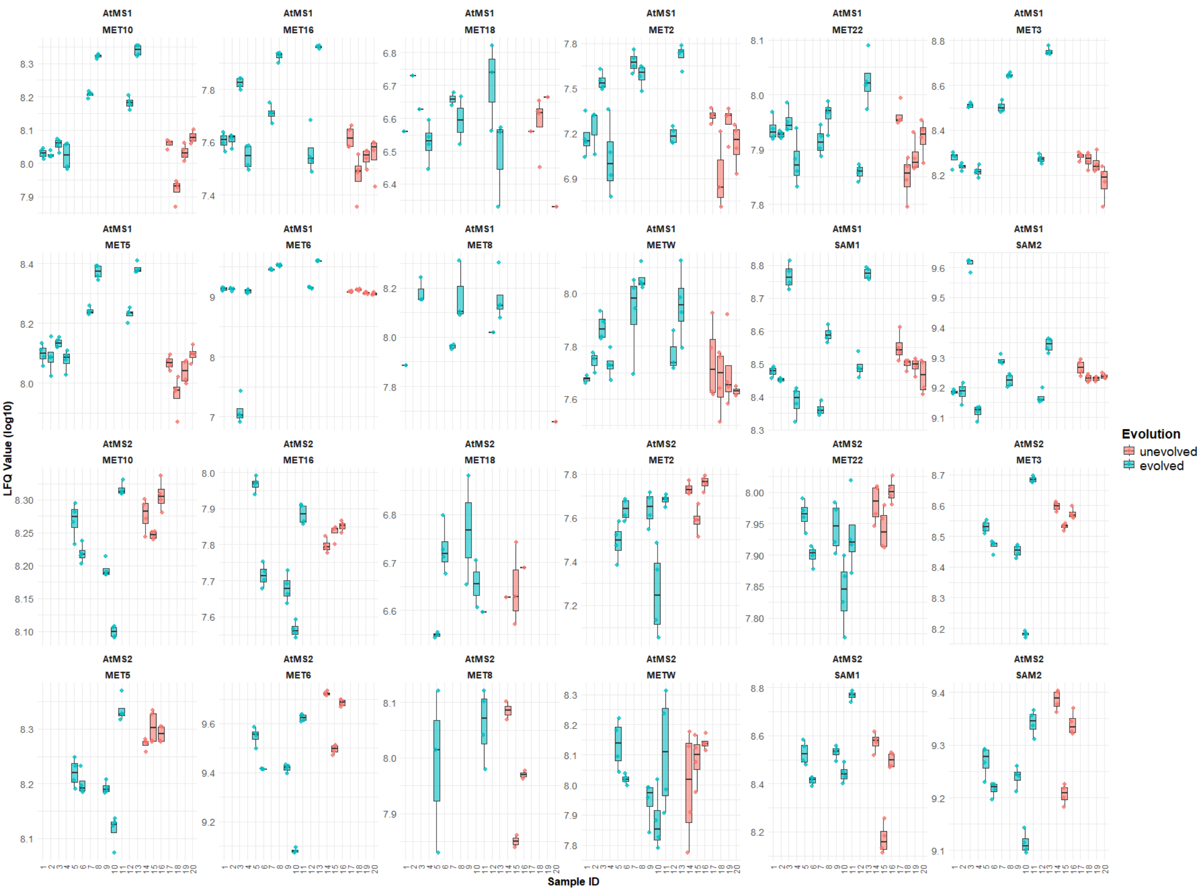

Figure S6. Analysis of Met related proteins in evolved and unevolved yeast populations. Untargeted proteomics analysis of both evolved and unevolved populations expressing AtMS1 or AtMS2. Expression of Met related proteins with corresponding LFQ values were obtained from mass spectrometry; n=4.

SUPPLEMENTAL TABLES

Table S1: Sequences of genes used in this study.

Name	Sequence	Source
AtMS1 WT	ATGGCTTCACACATTGTTGGATACCCACGTATGGGCCCTAAGAGAGAGCTC<br>AAGTTTGCATTGGAATCTTTCTGGGATGGTAAGAGCACTGCTGAGGATCTTC<br>AGAAGGTGTCTGCTGATCTCAGGTCATCCATCTGGAAACAGATGTCTGCCG<br>CTGGGACTAAGTTCATCCCTAGCAACACCTTTGCTCACTACGACCAGGTTCT<br>TGACACCACCGCCATGCTCGGTGCTGTTCCACCTAGGTATGGATACACTGGT<br>GGTGAGATCGGCCTTGATGTTTACTTCTCCATGGCTAGAGGAAATGCCTCTG<br>TGCCTGCCATGGAAATGACCAAGTGGTTCGACACCAACTACCATTACATCG<br>TCCCTGAGTTGGGCCCTGAGGTTAACTTCTCTTACGCATCCCACAAGGCGGT<br>GAATGAGTACAAGGAGGCCAAGGCTCTTGGTGTTGACACCGTCCCTGTACT<br>TGTTGGCCCAGTCTCTTACTTGCTGCTTTCCAAGGCTGCCAAGGGTGTTGAC<br>AAGTCATTCGAACTTCTTTCTCTTCTCCCTAAGATTCTCCCGATCTACAAGG<br>AAGTGATTACCGAGCTTAAGGCTGCTGGTGCCACCTGGATTCAGCTTGACG<br>AGCCTGTCCTTGTTATGGATCTTGAGGGTCAGAAACTCCAGGCCTTTACTGG<br>TGCCTATGCTGAACTTGAATCAACTCTTTCTGGTTTGAATGTTCTTGTCGAG<br>ACCTACTTCGCTGATATCCCTGCTGAGGCATACAAGACCCTAACCTCATTGA<br>AGGGTGTGACTGCCTTTGGATTTGATTTGGTTCGTGGCACCAAGACCCTTGA<br>TTTGGTCAAGGCAGGTTTCCCTGAGGGAAAGTACCTCTTTGCTGGTGTTGTT<br>GATGGAAGGAACATCTGGGCCAACGACTTTGCTGCGTCCCTAAGCACCTTG<br>CAGGCACTTGAAGGCATTGTTGGTAAAGACAAGCTTGTGGTCTCAACCTCCT<br>GCTCTCTTCTCCACACCGCTGTTGATCTTATCAATGAGACTAAGCTTGATGA<br>TGAAATCAAGTCATGGTTGGCGTTTGCTGCCCAGAAGGTCGTTGAAGTGAA<br>CGCTTTGGCCAAGGCTTTGGCTGGTCAGAAGGACGAGGCTCTTTTCTCTGCC<br>AATGCTGCGGCTTTGGCTTCAAGGAGATCTTCCCCAAGAGTCACCAACGAG<br>GGTGTCCAGAAGGCTGCTGCTGCTTTGAAGGGATCTGACCACCGTCGTGCA<br>ACCAATGTTAGTGCTAGGCTAGATGCTCAGCAGAAGAAGCTCAATCTCCCA<br>ATCCTACCAACCACAACCATTGGATCCTTCCCACAGACTGTAGAGCTCAGG<br>AGAGTTCGTCGTGAGTACAAGGCCAAAAAGGTCTCAGAGGAGGACTACGTT<br>AAAGCCATCAAGGAAGAGATCAAGAAAGTTGTTGACCTCCAAGAGGAACTT<br>GACATCGATGTTCTTGTCCACGGAGAGCCAGAGAGAAACGACATGGTTGAG<br>TACTTTGGTGAGCAGTTGTCTGGTTTTGCCTTCACTGCAAACGGATGGGTCC<br>AATCTTATGGATCTCGCTGTGTGAAGCCACCAGTTATCTATGGTGATGTGAG<br>CCGTCCCAAGGCAATGACCGTCTTCTGGTCCGCAATGGCTCAGAGCATGAC<br>CTCTCGCCCAATGAAGGGTATGCTTACTGGTCCCGTCACCATTCTCAACTGG<br>TCCTTTGTCAGGAACGACCAGCCCAGGCACGAAACCTGTTACCAGATCGCT<br>TTGGCCATCAAGGACGAAGTCGAGGATCTTGAGAAAGGTGGAATCGGTGTC<br>ATTCAGATTGATGAGGCTGCACTTAGAGAAGGACTACCACTCAGGAAATCC<br>GAGCACGCTTTCTACTTGGACTGGGCCGTCCACTCCTTCAGAATCACCAACT<br>GTGGAGTCCAAGACAGCACCCAGATCCACACTCACATGTGCTACTCCCACT<br>TCAATGACATCATACACTCCATCATCGACATGGATGCTGATGTCATCACCAT<br>TGAGAACTCCAGGTCTGATGAGAAGCTTCTTTCCGTGTTCCGTGAAGGAGTG<br>AAGTACGGTGCTGGAATCGGTCCAGGAGTCTACGACATCCACTCTCCAAGA<br>ATACCATCTTCTGAGGAAATCGCAGACAGGGTCAACAAGATGCTTGCTGTC<br>CTAGAGCAGAACATCCTTTGGGTTAACCCTGACTGTGGTCTCAAGACCCGTA<br>AGTACACCGAGGTCAAGCCTGCACTCAAGAACATGGTTGATGCGGCTAAGC<br>TCATCCGCTCCCAGCTCGCCAGTGCCAAGTGA	ABRC G13034
AtMS2 WT	ATGGCTTCCCACATTGTTGGATATCCACGTATGGGACCTAAGAGAGAGCTC<br>AAGTTTGCATTGGAGTCTTTCTGGGATGGCAAGAGCAGTGCCGATGATTTGC<br>AGAAGGTGTCTGCTGATCTCAGGTCTGATATCTGGAAACAGATGTCTGCTGC<br>TGGGATTAAGTATATCCCAAGCAACACCTTTTCTCATTATGACCAGGTGCTT<br>GACACCACCGCCATGCTTGGTGCTGTTCCATCTAGATATGGATTTACCAGTG<br>GTGAGATCGGTCTCGATGTTTACTTCTCCATGGCTAGAGGAAATGCCTCTGT<br>TCCAGCTATGGAGATGACCAAGTGGTTTGACACCAACTACCATTACATCGTC<br>CCAGAGTTGGGCCCTGAAGTGAAATTTTCTTACGCATCTCACAAGGCTGTCA<br>ATGAGTACAAGGAGGCCAAGGCTCTTGGTGTTGAGACCGTCCCTGTACTTG<br>TTGGCCCTGTCTCTTACTTGCTTCTTTCCAAGCTTGCTAAGGGTGTTGACAAG<br>TCATTTGATCTTCTCTCCCTTCTCCCCAAAATCCTCCCAGTTTACAAGGAAGT<br>CATTGCAGAGCTTAAGGCAGCTGGTGCCTCCTGGATTCAGCTTGATGAGCCT<br>CTCTTTGTCATGGATCTCGAGGGTCACAAACTCCAGGCTTTTAGCGGTGCCT<br>ATGCTGAGCTTGAATCAACTCTCTCTGGTCTGAATGTTCTTGTGGAGACCTA<br>CTTCGCTGATATCCCTGCTGAAGCATACAAGACCCTTACTTCCTTGAAGGGT<br>GTGACTGCCTTCGGATTTGATTTGGTTCGTGGCACCAAGACCATTGACTTGA	ABRC C104738

	TCAAGTCAGGTTTCCCACAGGGCAAGTACCTCTTTGCTGGTGTTGTTGACGG<br>AAGGAACATCTGGGCCAATGACCTCGCTGCCTCTCTCATCACCTTGCAGTCA<br>CTTGAGGGTGTTGTTGGTAAAGACAAGCTTGTGGTCTCAACCTCTTGCTCTC<br>TTCTCCACACTGCCGTTGACCTTATTAACGAGACTAAGCTTGATGCTGAAAT<br>CAAGTCGTGGCTAGCTTTTGCTGCCCAGAAGGTTGTTGAAGTTGACGCATTG<br>GCCAAGGCTTTGGCCGGTCAGACAAATGAGAGTTTCTTCACTGCCAACGCT<br>GACGCATTGTCTTCGAGGAGGTCTTCCCCAAGAGTCACCAATGAGTCTGTCC<br>AGAAGGCTGCTGCTGCTTTGAAGGGATCTGACCACCGCCGTACAACTGAAG<br>TTAGCGCAAGGCTAGATGCTCAGCAGAAGAAGCTTAACCTTCCAATCCTCC<br>CAACCACAACCATTGGATCCTTCCCACAGACCGTGGAACTCAGGAGAGTTC<br>GCCGTGAATACAAGGCCAAGAAAATCTCTGAAGAGGATTACGTCAAGGCCA<br>TCAAGGAAGAGATCAAGAAAGTTGTTGACATCCAAGAGGACCTTGACATTG<br>ATGTTCTTGTTCACGGAGAGCCTGAGAGAAACGACATGGTTGAGTACTTTG<br>GAGAGCAATTGTCAGGTTTCGCATTCACAGCAAACGGATGGGTGCAATCCT<br>ATGGATCTCGCTGTGTGAAGCCACCAGTTATCTATGGTGACGTGAGCCGCCC<br>CAAGCCAATGACAGTCTTCTGGTCCTCAACAGCTCAGAGCATGACCAAACG<br>TCCAATGAAGGGTATGCTTACAGGTCCAGTCACAATTCTCAACTGGTCTTTT<br>GTCAGAAACGACCAGCCCAGGCACGAAACCTGTTACCAGATCGCTTTGGCC<br>ATCAAAGATGAAGTGGAAGACCTCGAGAAAGGCGGTATTGGAGTCATTCAG<br>ATCGATGAAGCCGCACTTAGAGAAGGATTGCCTCTTAGGAAAGCCGAACAC<br>TCTTTCTACTTGGACTGGGCTGTTCACTCTTTCAGAATCACCAACTGTGGCG<br>TCCAAGACAGCACTCAGATTCACACTCACATGTGTTACTCAAACTTCAACG<br>ACATCATCCACTCAATCATTGACATGGACGCTGATGTCATCACCATTGAGAA<br>CTCTCGTTCAGACGAGAAGCTTCTCTCAGTGTTCCGTGAAGGAGTGAAGTAC<br>GGTGCAGGAATCGGTCCTGGTGTTTACGACATTCACTCTCCGAGAATACCAT<br>CCACAGATGAAATTGCAGACAGGATCAACAAGATGCTTGCGGTTCTTGAGC<br>AGAACATCTTGTGGGTTAACCCTGACTGTGGTCTGAAGACAAGGAAGTACA<br>CTGAGGTTAAACCAGCACTTAAAGCCATGGTTGACGCGGCTAAGCTTATCC<br>GCTCCCAGCTCGGTAGTGCCAAGTGA	
ScMET6	ATGGTTCAATCTGCTGTCTTAGGGTTCCCAAGAATCGGTCCAAACAGAGAA<br>TTAAAGAAGGCCACTGAAGGTTACTGGAACGGTAAAATCACTGTCGATGAA<br>TTATTCAAGGTCGGTAAGGATTTGAGAACTCAAAACTGGAAGTTGCAAAAG<br>GAGGCTGGTGTTGATATCATCCCATCCAATGACTTCTCCTTTTACGACCAAG<br>TTTTGGATTTGTCTTTGTTGTTCAATGTCATTCCAGACCGTTACACTAAGTAC<br>GATCTATCTCCAATCGACACTTTGTTTGCTATGGGTAGAGGTTTACAAAGAA<br>AGGCCACTGAAACTGAAAAGGCTGTCGACGTCACTGCTTTGGAAATGGTTA<br>AATGGTTCGACTCTAACTACCATTACGTTAGACCAACTTTCTCCAAGACCAC<br>TCAATTTAAGTTGAACGGCCAAAAGCCAGTTGACGAATTTTTGGAAGCCAA<br>GGAGTTAGGTATTCATACTAGACCTGTCTTGTTGGGTCCAGTTTCTTACTTAT<br>TCTTGGGTAAGGCTGACAAGGATTCTCTAGATTTGGAACCATTGTCCCTATT<br>GGAACAATTGTTGCCTCTATACACTGAAATCCTATCTAAATTGGCTTCTGCT<br>GGTGCCACTGAAGTTCAAATTGACGAACCTGTCTTAGTTTTGGACTTGCCTG<br>CCAACGCCCAAGCCGCCATTAAGAAGGCTTACACTTACTTCGGTGAACAAA<br>GCAATCTACCAAAGATTACTTTGGCTACTTACTTCGGTACCGTTGTCCCTAA<br>CTTAGACGCCATCAAGGGCTTGCCAGTTGCTGCCTTACACGTTGACTTTGTT<br>AGAGCTCCAGAACAATTTGATGAAGTCGTTGCCGCCATTGGTAACAAACAA<br>ACCTTGTCCGTTGGTATTGTTGATGGTAGAAACATTTGGAAGAATGATTTCA<br>AGAAGTCTTCCGCTATCGTTAACAAGGCTATTGAAAAGTTGGGTGCTGACA<br>GAGTCGTTGTTGCCACTTCTTCTTCTCTATTGCACACACCAGTTGATTTGAAC<br>AACGAAACCAAGTTGGACGCTGAAATCAAGGGCTTTTTCTCTTTCGCCACTC<br>AAAAATTGGATGAAGTTGTTGTGATCACCAAGAACGTTTCCGGTCAAGACG<br>TTGCTGCTGCCCTAGAAGCTAACGCTAAATCTGTTGAATCCAGAGGTAAATC<br>CAAGTTTATCCACGATGCTGCCGTTAAGGCCAGAGTTGCCTCTATCGACGAA<br>AAAATGTCTACTAGAGCAGCTCCATTTGAACAAAGATTGCCTGAACAACAA<br>AAAGTCTTCAACTTGCCATTGTTCCCAACAACAACTATTGGTTCCTTCCCTC<br>AAACCAAGGACATCAGAATTAACAGAAACAAATTCAACAAGGGCACCATC<br>TCTGCTGAAGAATATGAAAAATTCATCAATTCTGAAATTGAAAAGGTCATC<br>AGATTCCAAGAAGAAATTGGTTTGGATGTCTTAGTCCACGGTGAACCAGAA<br>AGAAACGATATGGTTCAATACTTCGGTGAACAAATCAACGGTTATGCTTTC<br>ACTGTTAACGGTTGGGTTCAATCTTACGGTTCCAGATATGTCAGACCACCAA<br>TTATTGTTGGTGACTTGTCCAGACCAAAGGCTATGTCCGTCAAGGAATCTGT<br>TTACGCTCAATCCATCACTTCTAAGCCAGTAAAGGGTATGTTGACTGGTCCA<br>ATTACCTGTTTGAGATGGTCTTTCCCAAGAGACGATGTCGACCAAAAAACTC<br>AAGCTATGCAATTAGCTTTGGCTTTGAGAGATGAAGTCAATGATTTGGAAG<br>CTGCCGGTATCAAGGTTATCCAAGTTGATGAACCAGCTTTAAGAGAAGGTT<br>TACCATTGAGAGAAGGTACTGAGAGATCTGCTTACTACACCTGGGCTGCCG<br>AAGCTTTCAGAGTTGCTACTTCTGGTGTTGCTAACAAGACTCAAATACACTC<br>TCATTTCTGTTACTCTGACTTGGATCCAAACCATATCAAGGCTTTGGATGCT	Amplified from gDNA of Saccharomyces cerevisiae BY4742 (Euroscarf)

	GATGTTGTTTCCATCGAATTCTCTAAGAAGGACGATGCTAACTACATTGCTG AATTCAAAAACTATCCAAACCACATTGGTCTAGGTTTATTCGATATTCATTC TCCAAGAATTCCATCAAAGGATGAATTTATCGCCAAGATTTCAACCATCTTG AAGAGCTACCCAGCTGAAAAGTTCTGGGTTAACCCAGATTGTGGTTTGAAG ACTAGAGGCTGGGAAGAAACTAGATTGTCTTTGACTCATATGGTCGAAGCC GCCAAGTATTTCCGTGAACAATACAAGAATTAA	

Table S2: Primers used for amplification and sequencing.

Name	Sequence	Template	Purpose
AtMS1_NP_F	GGTGGTGAATTCAATGGCTTCACACATT GTTGGATACCC	Arabidopsis thaliana METS1	Cloning into ArEc-TDH3
AtMS1_R	GGTGGTGCATGCTCACTTGGCACTGGC GAGCTG	Arabidopsis thaliana METS1	Cloning into ArEc-TDH3 and GR-306MP
AtMS1_P1_F	GGTGGTATGCATGCTTCACACATTGTTG GATACCCAC	Arabidopsis thaliana METS1	Cloning into GR-306MP
AtMS2_NP_F	GAATACGGGTTCGAATTCATGGCTTCCC ACATTGTTGGATATCC	Arabidopsis thaliana METS2	Cloning into ArEc-TDH3
AtMS2_ R	GCTGGTGATCACGCATGCTCACTTGGC ACTACCGAGCTGG	Arabidopsis thaliana METS2	Cloning into ArEc-TDH3 and GR-306MP
AtMS2_P1_F	GAATACGGGTTCATGCATGCTTCCCACA TTGTTGGATATCCAC	Arabidopsis thaliana METS2	Cloning into GR-306MP
ScMET6_P1_F	GAATACGGGTTCATGCATGTTCAATCT GCTGTCTTAGGGTTCC	Saccharomyces cerevisiae MET6	Cloning into GR-306MP
ScMET6_P1_R	GCTGGTGATCACGCATGCTTAATTCTTG TATTGTTCACGGAAGTACTTGG	Saccharomyces cerevisiae MET6	Cloning into GR-306MP
MET6_Seq1	GAAGCCAAGGAGTTAGGTATTCATAC	Saccharomyces cerevisiae MET6	Sequencing
MET6_Seq2	CTATTGAAAAGTTGGGTGCTGACAGAG	Saccharomyces cerevisiae MET6	Sequencing
MET6_Seq3	CGGTTGGGTTCAATCTTACGGTTC	Saccharomyces cerevisiae MET6	Sequencing
AtMS2_Seq1	CTGTCTCTTACTTGCTTCTTTCCAAGC	Arabidopsis thaliana METS2	Sequencing
AtMS2_Seq2	CTCTCATCACCTTGCAGTCACTTG	Arabidopsis thaliana METS2	Sequencing
AtMS2_Seq3	CAATGACAGTCTTCTGGTCCTCAAC	Arabidopsis thaliana METS2	Sequencing
AtMS1_Seq1	GAGTACAAGGAGGCCAAGGCTC	Arabidopsis thaliana METS1	Sequencing
AtMS1_Seq2	GAAATCAAGTCATGGTTGGCGTTTGC	Arabidopsis thaliana METS1	Sequencing
AtMS1_Seq3	CAATGAAGGGTATGCTTACTGGTC	Arabidopsis thaliana METS1	Sequencing
Rec_p1_F	GAGATTATTGGAAGATTAGTACGTCTCC	p1 plasmid	Amplification
polyA_R	GAAAAGTATGACCTAATTGACTCCGG	p1 plasmid	Amplification
P1_Rec_F	GTCTATTTTACACTTTTGACCTATAAGTC	p1 plasmid	Sequencing

Table S3: Peptide sequences and mass spectrometry analysis parameters used for targeted selected reaction monitoring analysis of AtMS1 and AtMS2.

Protein	Peptide	Precursor Mz	Precursor Charge	Product Mz	Product Charge	Fragment Ion	Retention Time
AtMS1	STAEDLQK	446.224539	2	703.3621	1	y6	1.65
AtMS1	STAEDLQK	446.224539	2	632.32498	1	y5	1.65
AtMS1	STAEDLQK	446.224539	2	503.28239	1	y4	1.65
AtMS1	STAEDLQK	446.224539	2	275.17138	1	y2	1.65
AtMS1	STAEDLQK	446.224539	2	189.08698	1	b2	1.65
AtMS1	QMSAAGTK	397.19727	2	534.2882	1	y6	0.62
AtMS1	QMSAAGTK	397.19727	2	447.25617	1	y5	0.62
AtMS1	QMSAAGTK	397.19727	2	376.21906	1	y4	0.62
AtMS1	QMSAAGTK	397.19727	2	305.18195	1	y3	0.62
AtMS1	AGFPEGK	353.181946	2	634.3195	1	y6	3.09
AtMS1	AGFPEGK	353.181946	2	430.22962	1	y4	3.09
AtMS1	AGFPEGK	353.181946	2	333.17686	1	y3	3.09
AtMS1	AGFPEGK	353.181946	2	204.13427	1	y2	3.09
AtMS1	VSEEDYVK	484.732197	2	869.3887	1	y7	4.57
AtMS1	VSEEDYVK	484.732197	2	782.35668	1	y6	4.57
AtMS1	VSEEDYVK	484.732197	2	653.31408	1	y5	4.57
AtMS1	VSEEDYVK	484.732197	2	409.24455	1	y3	4.57
AtMS1	VSEEDYVK	484.732197	2	187.10772	1	b2	4.57
AtMS1	IPSSEEIADR	558.78021	2	1003.4691	1	y9	6.32
AtMS1	IPSSEEIADR	558.78021	2	906.41632	1	y8	6.32
AtMS1	IPSSEEIADR	558.78021	2	819.38429	1	y7	6.32
AtMS1	IPSSEEIADR	558.78021	2	603.30967	1	y5	6.32
AtMS1	IPSSEEIADR	558.78021	2	502.23818	2	y9	6.32
AtMS1	NMVDAAK	374.686538	2	404.21397	1	y4	2.19
AtMS1	NMVDAAK	374.686538	2	289.18703	1	y3	2.19
AtMS1	NMVDAAK	374.686538	2	218.14992	1	y2	2.19
AtMS2	SSADDLQK	432.208889	2	776.37847	1	y7	1.19
AtMS2	SSADDLQK	432.208889	2	689.34645	1	y6	1.19
AtMS2	SSADDLQK	432.208889	2	618.30933	1	y5	1.19
AtMS2	SSADDLQK	432.208889	2	147.1128	1	y1	1.19
AtMS2	SSADDLQK	432.208889	2	175.07133	1	b2	1.19
AtMS2	QMSAAGIK	403.215463	2	546.32459	1	y6	3.45
AtMS2	QMSAAGIK	403.215463	2	388.25545	1	y4	3.45
AtMS2	QMSAAGIK	403.215463	2	317.21833	1	y3	3.45
AtMS2	EVIAELK	401.239461	2	573.36064	1	y5	7.45
AtMS2	EVIAELK	401.239461	2	460.27658	1	y4	7.45
AtMS2	EVIAELK	401.239461	2	389.23946	1	y3	7.45
AtMS2	SGFPQGK	360.687396	2	429.24561	1	y4	2.13
AtMS2	SGFPQGK	360.687396	2	332.19285	1	y3	2.13
AtMS2	SGFPQGK	360.687396	2	204.13427	1	y2	2.13
AtMS2	SGFPQGK	360.687396	2	145.06077	1	b2	2.13
AtMS2	VVEVDALAK	472.276575	2	844.47746	1	y8	8.85
AtMS2	VVEVDALAK	472.276575	2	745.40905	1	y7	8.85
AtMS2	VVEVDALAK	472.276575	2	616.36645	1	y6	8.85

AtMS2	VTNESVQK	452.740356	2	805.40502	1	y7	0.81
AtMS2	VTNESVQK	452.740356	2	704.35734	1	y6	0.81
AtMS2	VTNESVQK	452.740356	2	275.17138	1	y2	0.81
AtMS2	TTEVSAR	382.200867	2	561.2991	1	y5	0.83
AtMS2	TTEVSAR	382.200867	2	432.25651	1	y4	0.83
AtMS2	TTEVSAR	382.200867	2	333.18809	1	y3	0.83
AtMS2	ISEEDYVK	491.740022	2	869.3887	1	y7	5.74
AtMS2	ISEEDYVK	491.740022	2	782.35668	1	y6	5.74
AtMS2	ISEEDYVK	491.740022	2	409.24455	1	y3	5.74
AtMS2	ISEEDYVK	491.740022	2	246.18122	1	y2	5.74
AtMS2	IPSTDEIADR	558.78021	2	1003.4691	1	y9	7.35
AtMS2	IPSTDEIADR	558.78021	2	906.41632	1	y8	7.36
AtMS2	IPSTDEIADR	558.78021	2	819.38429	1	y7	7.36
AtMS2	IPSTDEIADR	558.78021	2	502.23818	2	y9	7.36
AtMS2	AMVDAAK	353.183631	2	503.28239	1	y5	1.99
AtMS2	AMVDAAK	353.183631	2	404.21397	1	y4	1.99
AtMS2	AMVDAAK	353.183631	2	289.18703	1	y3	1.99
